# Disabling Orthostatic Headache after Penetrating Stonemason Pencil Injury to the Sacral Region

**DOI:** 10.1155/2015/623405

**Published:** 2015-10-26

**Authors:** Carlo Brembilla, Luigi Andrea Lanterna, Paolo Gritti, Emanuele Costi, Gianluigi Dorelli, Elena Moretti, Claudio Bernucci

**Affiliations:** ^1^Department of Neurosurgery, Pope John XXIII Hospital, OMS Square No. 1, 24100 Bergamo, Italy; ^2^Department of Anesthesia and Intensive Care, Pope John XXIII Hospital, OMS Square No. 1, 24100 Bergamo, Italy

## Abstract

Penetrating injuries to the spine, although less common than motor vehicle accidents and falls, are important causes of injury to the spinal cord. They are essentially of two varieties: gunshot or stab wounds. Gunshot injuries to the spine are more commonly described. Stab wounds are usually inflicted by knife or other sharp objects. Rarer objects causing incidental spinal injuries include glass fragments, wood pieces, chopsticks, nailguns, and injection needles. Just few cases of penetrating vertebral injuries caused by pencil are described. The current case concerns a 42-year-old man with an accidental penetrating stonemason pencil injury into the vertebral canal without neurological deficit. After the self-removal of the foreign object the patient complained of a disabling orthostatic headache. The early identification and treatment of the intracranial hypotension due to the posttraumatic cerebrospinal fluid (CSF) sacral fistulae were mandatory to avoid further neurological complications. In the current literature acute pattern of intracranial hypotension immediately after a penetrating injury of the vertebral column has never been reported.

## 1. Introduction

Penetrating injury is the third most frequent cause of spinal injuries in adults, only surpassed by traffic accidents and falls [1]. Gunshot wounds and knife stabbings account for the majority of penetrating spinal injuries [2, 3]. Other rare objects usually cause incidental penetrating spinal injury [4–6].

The current case concerns a 42-year-old man with an accidental penetrating stonemason pencil injury to the sacral region. The man was referred to the attention of Pope John XXIII Hospital Emergency Department in Bergamo with a disabling orthostatic headache.

## 2. Case Report

**Figure 1:**
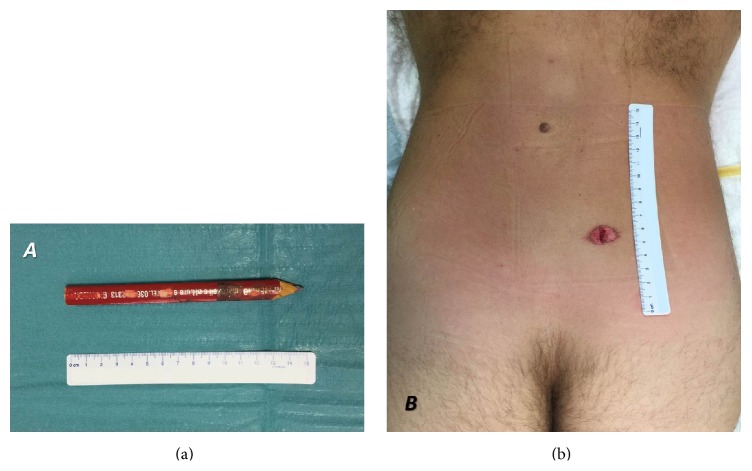
The wound caused by the penetrating object in the lumbosacral region (b), slightly to the right of the midline at the level of S1, and the penetrating object: a stonemason pencil (a).

A 42-year-old man was referred to the attention of Pope John XXIII Hospital Emergency Department in Bergamo after falling while working. The man, a stonemason, fell walking backwards in the dockyard. A stonemason pencil (Figure 1(a)) carried in his work pouch stabbed him in the lumbosacral region. After the falling, the man removed from himself the foreign body. Immediately he complained of severe lumbosacral pain. After few minutes there appeared a disabling orthostatic headache that forced the man into supine position.

**Figure 2:**
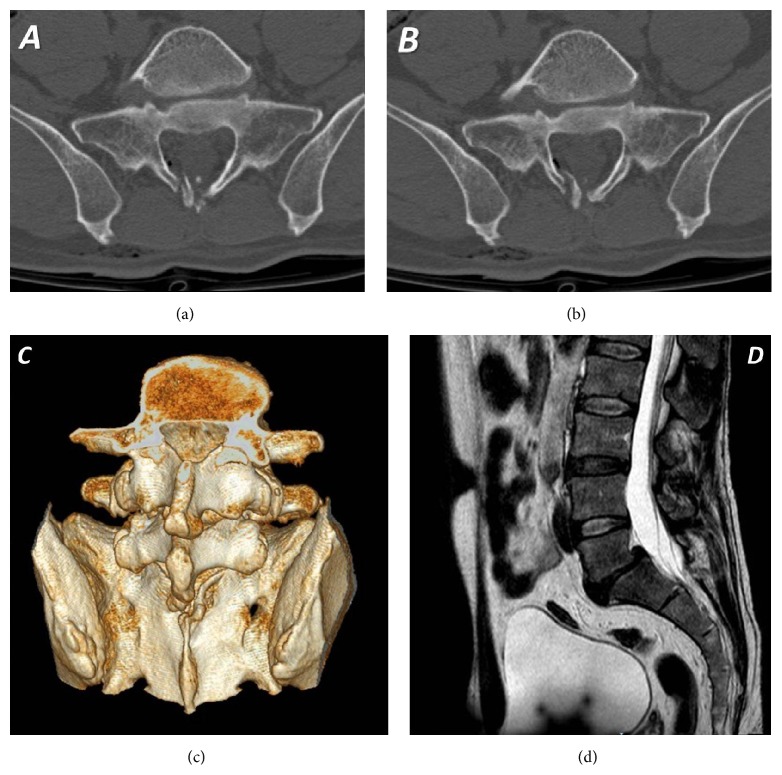
Lumbosacral CT scan ((a), (b), (c)) and RMI (d) showing a multifragmented fracture of the S1 lamina, with air bubbles in the epidural space.

**Figure 3:**
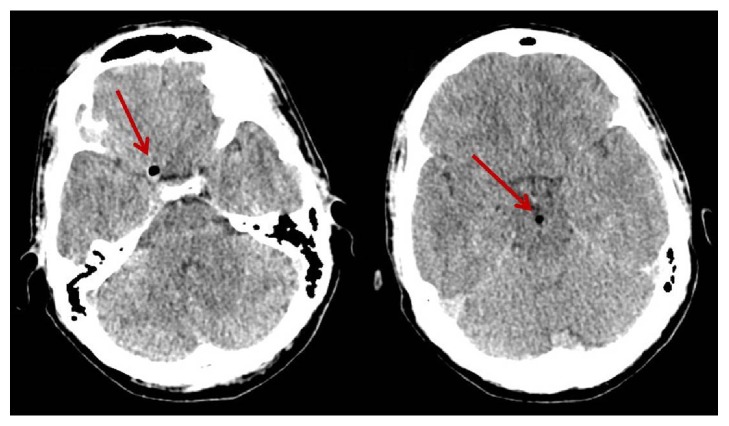
Cranial CT scan showing pneumocephalus.

On admission the inspection revealed a wound in the lumbosacral region (Figure 1(b)), slightly to the right of the midline, with serohematic fluid attributable to cerebrospinal fluid (CSF) leakage. Physical examination revealed severe contracture of the lumbosacral muscles. At the neurological examination no deficit was detected on lower limbs. Bowel and bladder function were intact. The patient could not stand on his feet because of the orthostatic headache. The lumbosacral pain score on a visual analog scale (VAS) of 0–100, with 0 representing no pain and 100 representing severe pain, was 70; the headache, in standing position, was quantified 80. A complete blood count showed mild normocytic anemia and moderate leukocytosis. A lumbosacral CT scan showed a multifragmented fracture of the S1 lamina, with air bubbles in the epidural space (Figures 2(a), 2(b), and 2(c)). A lumbosacral RMI confirmed the presence of the S1 lamina fracture and the contusion of the lumbosacral muscles along the foreign object trajectory (Figure 2(d)). The neuroradiological exams also showed incidentally L5-S1 isthmic spondylolisthesis. In order to investigate the headache, a cranial CT scan showed diffused pneumocephalus (Figure 3), without dislocation of the cerebellar tonsils. In light of the patient's history as well as findings on physical examination and imaging studies, a diagnosis of intracranial hypotension due to posttraumatic CSF lumbosacral fistulae was made.

**Figure 4:**
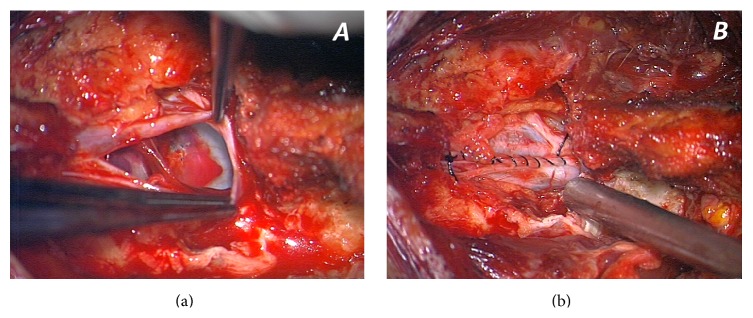
Intraoperative pictures from the high resolution microscope. (a) The dural laceration, with sacral roots contused, and the impact point of the penetrating object in the anterior part of the dural sac, at the level of the posterior wall of S1 vertebral body; in the middle of the contusion some little graphite pigments from the pencil tip can be seen. (b) The watertight closure of the dural sac.

The patient underwent a surgical intervention. A linear skin incision on the midline at S1 level was made to expose the multifragmented fracture of the S1 lamina. After the removal of the bone fragments, the exposition of the vertebral canal was completed with a minimal laminectomy of S1. The dura mater was lacerated for about 1.5 centimeters (Figure 4(a)). No residual part of the pencil was detected into the dural sac. A watertight closure of the dural sac was achieved (Figure 4(b)). The entry point of the foreign body, after a surgical toilette, and the surgical incision were both closed in layers.

After surgery no neurological deficit appeared. The patient walked on postoperative day 3 with an elastic lower back support. A 7-day course of antibiotic was given for prophylaxis: amoxicillin-clavulanate 850 mg/125 mg (Augmentin) three times daily. A postoperative cranial CT scan showed an improvement of the pneumocephalus. After one month the patient reported no pain and episodes of fever. A new cranial CT scan showed the resolution of the pneumocephalus. After 3 months still no fever episodes and pain were reported.

## 3. Discussion

In adults, penetrating injuries constitute the third most frequent cause of spinal cord injuries [1]. Surpassed only by traffic accidents and falls, they account for four to seven cases per million persons per year [2]. Penetrating injuries are essentially of two varieties: gunshot or stab wounds. Gunshot injuries to the spine are more commonly described and are associated with a higher incidence of neurological damage [1–3]. Stab wounds are usually inflicted by knife or other sharp, knife-like objects. Commonly, the wound is inflicted from behind during an assault, and it results in an incomplete spinal cord injury [7].

Rarer objects causing accidental spinal injuries include glass fragments, wood pieces, chopsticks, nailguns, and injection needles [4–6]. In the current literature just few cases of penetrating vertebral injuries caused by pencil are described [8]. Between these only two cases regard the penetration of a pencil into the spinal canal, both being in pediatric patients. In 2006 Piqueras et al. [9] reported the case of a 10-year-old boy who sustained an injury to the cauda equina as a result of the accidental penetration during a fall of a wooden pencil into the spinal canal at the L5-S1 level. After neuroimaging evaluation, the foreign body was removed and the wound was repaired. As a precaution, the child was treated with antibiotics. After a follow-up period of 1 year, the boy's neurological deficits had completely resolved. Still in 2006 Ramaswamy et al. [10] reported the case of a 12-year-old boy that suffered a penetrating injury from a pencil contained in his coat pocket during a rough tumble with his friends. The pencil penetrated the posterior wall of his chest on the left side, extended through the dorsal paraspinal soft tissues into the neuroforaminal canal on the left at T12-L1 level, and across the midline reached the right neuroforaminal canal. The penetrating injury gave a complete spinal cord damage. After neuroimaging evaluation, the child underwent a surgical operation to decompress the spinal canal and remove the foreign object. In the postoperative time the patient did not improve the neurological deficit.

The initial principles in the management of penetrating intraspinal injuries should include a meticulous neurologic examination and the administration of prophylactic antibiotic agents. Particular attention should be paid and priority given to eventually life-threatening visceral and vascular injuries [1–11].

Radiograph and CT of the spine are essential to demonstrate bony injuries, retained foreign bodies, and signs of spinal instability [12]. Radiologic investigation of the vessel anatomy should be performed if the trajectory of the blade predicts damage to any important artery. In most cases, the foreign body is metal, plastic, or glass, all of which are usually easily detected on conventional radiograph [12]. However, wooden foreign bodies are difficult to detect with plain radiograph; therefore, their diagnosis is often missed or delayed. A CT scan is usually performed in cases where entry of the foreign bodies is suspected. But a wooden foreign body is known to initially be recognized as a hypodense image on the CT scan and consequently diagnosed as air. Therefore, MRI is advised to be used in adjunction with CT. MRI scan can be very useful to demonstrate and localize the foreign body and also to exclude any intra- or extradural hematoma or contusion in the cord or cauda equine [13].

The objective of the surgical treatment is to decompress the spinal cord, remove the foreign body, prevent cerebrospinal fluid leakage, and eventually stabilize the vertebral column [14–16]. Some authors emphasize surgery for removal of the retained object to avoid progressive neurological deterioration, especially if wood is involved, as wood in particular can be irritant to tissue. In 1993 Sawar et al. [17] reported a case in which penetrating injury to the midthoracic spine was caused by a piece of wood in a patient involved in a road traffic accident. The patient underwent a surgical intervention 3 years after the injury for indurated and fluctuant swelling over the penetrating injury. The wound had healed completely after the removing of a wood piece from the spinal canal and the toilette of the granulation tissue around. In 2000 Lunawat and Taneja [18] reported the case of 18-year-old boy who presented with weakness in his lower limbs and had an upper motor neuron lesion at the D12-L1 level. At laminectomy two stone-like objects were found which proved to be bundles of tiny pieces of wood. They are thought to have entered the cord through an abdominal penetrating injury sustained six years previously.

The current case concerns 42-year-old man with an accidental penetrating stonemason pencil injury into the vertebral canal without neurological deficit. After the removal of the foreign object the subsequent posttraumatic CSF fistulae gave an acute clinical pattern of intracranial hypotension. An early identification and treatment of the intracranial hypotension was mandatory to avoid further neurological complications. In the current literature acute pattern of intracranial hypotension due to posttraumatic CSF fistulae by penetrating object into the spinal canal has never been reported.
